# Smart Technology–Assisted Patient-Centered Management in Venous Thromboembolism: Pilot Study on Anticoagulation Adherence

**DOI:** 10.2196/75508

**Published:** 2026-04-02

**Authors:** Zheqi Zhang, Zhigeng Jin, Hao Wang, Hui Zhang, Binbin Liu, Hong Wang, Zhenguo Zhai, Yutao Guo

**Affiliations:** 1 Medical School of Chinese People’s Liberation Army, Chinese PLA General Hospital, No.28 Fuxing Road, Haidian District, Beijing, 100853, China; 2 Department of Pulmonary Vascular and Thrombotic Disease, the Sixth Medical Center of Chinese PLA General Hospital, Chinese PLA General Hospital, No.6 Fucheng Road, Haidian District, Beijing, 100048, China, 86 1 381 002 1492; 3 National Clinical Research Center for Geriatric Diseases, the Second Medical Center, Chinese PLA General Hospital, Beijing, China; 4 Department of Pulmonary and Critical Care Medicine, Center of Respiratory Medicine of China-Japan Friendship Hospital, China-Japan Friendship Hospital, Beijing, China

**Keywords:** anticoagulation management, venous thromboembolism, medication adherence, smart technology, patient-centered

## Abstract

**Background:**

Achieving optimal adherence to anticoagulation therapy is a major challenge in the management of venous thromboembolism (VTE). Mobile health (mHealth) technologies may offer a scalable approach to supporting medication adherence and self-management.

**Objective:**

This pilot study aimed to assess the feasibility and preliminary impact of a smart technology–assisted, patient-centered care mHealth app for managing VTE (mVTEA) on short-term anticoagulation adherence among patients with VTE or at moderate-to-high risk of VTE.

**Methods:**

Baseline medication adherence and beliefs were assessed using the Chinese versions of the 8-item Morisky Medication Adherence Scale and the Beliefs about Medicines Questionnaire–Specific to characterize baseline status only. The primary outcome was perfect adherence at 1 month, assessed through structured telephone interviews, outpatient visits, and the mVTEA physician-patient communication module. During follow-up, researchers verified current medication regimens, recorded missed doses, assessed therapy continuation, and whenever possible, confirmed adherence through pharmacy refill records or remaining medication packaging. Secondary outcomes included the mVTEA check-in rate and clinical safety events (VTE recurrence, major bleeding per International Society on Thrombosis and Haemostasis criteria, VTE-related hospitalizations, VTE-related rehospitalizations, all-cause mortality).

**Results:**

In total, 45 participants completed the study (mean age 60.80, SD 15.20 years; n=16, 36% female). Baseline 8-item Morisky Medication Adherence Scale scores indicated suboptimal adherence (mean 6.24, SD 1.80), with 29% (13/45) classified as good adherence and 71% (32/45) as moderate or poor adherence. The primary contributors to nonadherence were forgetting to take medication. Baseline Beliefs about Medicines Questionnaire–Specific scores showed stronger beliefs in medication necessity than concerns (17.58, SD 2.52 vs 14.56, SD 3.34; *P*<.001; necessity-concerns differentials: 3.02, SD 4.60). At 1-month follow-up, all 45 participants achieved perfect adherence, and 80% (n=36) used the mVTEA check-in feature. Participants who engaged in check-ins demonstrated markedly more favorable necessity-concerns differentials profiles (29/36, 81% vs 0/9, 0%; *P*<.001). No VTE events, major bleeding, or other adverse outcomes were reported.

**Conclusions:**

This pilot study supports the feasibility and acceptability of the mVTEA and provides preliminary signals that it may support short-term anticoagulation adherence. Larger randomized trials with longer follow-up and objective adherence measures are warranted, along with efforts to address the digital divide to ensure equitable access to mHealth-based anticoagulation support.

## Introduction

Venous thromboembolism (VTE), encompassing deep vein thrombosis and pulmonary embolism (PE), remains a leading cause of preventable cardiovascular morbidity and mortality globally, with an annual incidence ranging from 100 to 200 per 100,000 population [1,2]. In China, driven by an aging population and improved diagnostic capabilities, the incidence of VTE is progressively increasing, posing a significant public health challenge [3]. Beyond acute lethality, VTE is associated with debilitating long-term complications, including chronic thromboembolic pulmonary hypertension and postthrombotic syndrome, which severely impair quality of life and impose substantial economic burdens on health care systems [2]. Moreover, extended pharmacological thromboprophylaxis (ie, continued anticoagulation postdischarge) is frequently warranted for surgical patients at high VTE risk (eg, major orthopedic procedures, abdominal cancer surgeries) and specific high-risk medical populations (eg, active malignancy, prior VTE history) [4]. Consequently, optimizing comprehensive VTE management holds substantial clinical significance for improving patient outcomes and conserving health care resources.

Anticoagulation therapy is the cornerstone of VTE management [5]. However, maintaining anticoagulation adherence remains a critical challenge. Suboptimal patient adherence is multifactorial, stemming from patient-level factors (eg, inadequate disease awareness, fear of bleeding, forgetfulness), medication-related barriers (eg, cost, need for monitoring with warfarin), and follow-up management issues (eg, insufficient physician-patient communication, poor coordination among multidisciplinary care teams) [6-8]. Various strategies have been implemented to improve adherence, including systematic health education to enhance patient knowledge [9], cluster nursing interventions to optimize warfarin adherence and knowledge [10], and specialized anticoagulation clinics [11]. However, these approaches generally face limitations such as high human resource dependency, limited coverage, and challenges in implementing large-scale personalized interventions.

The digital health era offers unprecedented opportunities to address these challenges. Through mobile health (mHealth) apps, patients can conveniently record symptoms, monitor vital signs, and report medication behaviors, facilitating a shift from passive treatment acceptance to active participation in management [12,13]. While some mHealth apps exist for general anticoagulation management [14,15], there is a paucity of evidence-based platforms specifically designed for VTE patients that integrate dynamic risk assessment, personalized behavioral nudges, and patient-provider communication. To address this gap, we have developed a smart technology–assisted, patient-centered care mHealth app for managing VTE (mVTEA) [16]. This novel application can leverage real-time data to dynamically assess thrombosis/bleeding risks and deliver tailored interventions, aiming to establish a “partnership-style” interactive relationship and enhance patients’ self-efficacy and treatment engagement.

This pilot study aims to evaluate the feasibility and preliminary efficacy of the mVTEA in improving short-term anticoagulation among patients with VTE or at moderate-to-high risk of VTE. The findings will provide essential parameters for designing future definitive randomized controlled trials.

## Methods

### Study Design and Setting

This was a single-center, prospective, single-arm pilot study conducted at the Sixth Medical Center of the Chinese People’s Liberation Army General Hospital between August 25, 2023, and December 20, 2023. The hospital is a tertiary medical institution integrating clinical care, teaching, and research. The study was designed to evaluate the feasibility and preliminary efficacy of the mVTEA on anticoagulation adherence among patients with or at risk of VTE over a 1-month follow-up period. Both the design and reporting of this study comply with the CONSORT (Consolidated Standards of Reporting Trials) 2010 statement extension for pilot and feasibility trials [17] (Checklist 1). This study was registered with the Chinese Clinical Trial Registry (ChiCTR2200063206).

### Participants and Recruitment

Potentially suitable candidates for the study were initially screened from the hospitalized patients in the Department of Pulmonary Vascular and Thrombotic Disease through the clinical decision support system for VTE risk assessment and integrated care at the Sixth Medical Center of the Chinese People’s Liberation Army General Hospital [18]. Based on the initial screening list, a final identification of whether the patient met the study recruitment needs was made on the day the patient was discharged from the hospital. Inclusion criteria were (1) age ≥18 years; (2) diagnosed with confirmed VTE (deep vein thrombosis or PE) or identified as being at moderate-to-high risk of VTE based on the Padua Prediction Score (score ≥4) or Caprini Risk Assessment Model (score ≥2); (3) currently prescribed anticoagulant medication for VTE treatment or prophylaxis for a planned duration of ≥1 month; (4) able to understand the study procedures and willing to provide informed consent; (5) proficient in the use of a smartphone and willing to download and use the mVTEA. Exclusion criteria included (1) severe cognitive impairment or mental disorders precluding the use of the mVTEA; (2) lack of access to a smartphone; and (3) participation in other interventional clinical trials.

Patients fulfilling these requirements and expressing interest in participation received a comprehensive, in-person explanation of the study objectives, procedures, potential benefits, and possible risks. Those who agreed to enroll were provided with a paper-based informed consent form for signature at the same time. The process was conducted with full respect for each participant’s preferences and autonomy. In addition, we assured that the rights of participants to withdraw from the study would be upheld throughout all phases of the clinical trial.

### Intervention

#### Overview

Patients enrolled in the study were registered with mVTEA via smartphone. The registration process collected essential demographic and clinical information, including name, gender, age, height, weight, hospitalization department, use of anticoagulants, and contact details. During the inpatient period, departmental health care staff provided VTE health education as part of the institution’s standard protocol for VTE prevention and management. A tailored VTE prevention and treatment plan was formulated by the patient’s supervising physician. Prior to discharge, patients were asked to complete the Chinese version of the 8-item Morisky Medication Adherence Scale (MMAS-8) [19] and the Beliefs about Medication Questionnaire–Specific (BMQ-Specific). Upon discharge, the mVTEA system commenced sending reminders for anticoagulant medication administration according to the prescribed regimen. Patients were instructed to log their medication adherence using mVTEA on the day of administration or on subsequent days for additional entries. The system also provided weekly feedback on adherence data. A follow-up assessment was conducted on the 30th day postdischarge, either via telephone or in a clinical setting.

The Chinese versions of MMAS-8 and BMQ-Specific used in this study have demonstrated acceptable psychometric properties in several chronic disease populations in China [20-24]. However, neither instrument has been formally validated in VTE populations. Although some concerns have been raised regarding the MMAS-8 measurement’s precision and structural stability across various clinical contexts, this scale remains widely used in medication adherence research due to its brevity, feasibility, and comparability across chronic diseases [20-22,25]. Given the exploratory and pilot nature of this study, MMAS-8 and BMQ-Specific were used as preliminary measures to characterize baseline adherence and medication beliefs. Their use enabled standardized and comparable assessments across participants and facilitated the identification of attitudinal barriers prior to the initiation of mVTEA-supported management.

### The Chinese Version of MMAS-8

Medication adherence was tested using the Chinese version of the 8-item MMAS-8 (Multimedia Appendix 1). Response choices were “yes” or “no” for items 1 to 7, and Item 8 had a 5-point Likert response scale. Each “no” response was rated as 1 and each “yes” response was rated as 0 except for item 5, in which each “yes” response was rated as 1 and each “no” response was rated as 0. For Item 8, the code (0‐4) had to be standardized by dividing the result by 4 to calculate a summated score. Total scores on the MMAS-8 ranged from 0 to 8, with scores of 8 reflecting high adherence, 7 or 6 reflecting medium adherence, and<6 reflecting low adherence [20]. The psychometric properties of the Chinese version of MMAS-8 are favorable, with high internal consistency (Cronbach *α*=.817) and excellent test-retest reliability (intraclass correlation=0.947) [20]. Permission to use the MMAS-8 was obtained from the copyright holder and the scale was used in accordance with the licensing agreement.

### The Simplified Chinese Version of BMQ-Specific

The simplified Chinese version of BMQ-Specific was used to evaluate individuals’ perceptions of taking prescribed anticoagulants (Multimedia Appendix 2). It consisted of 10 items: 5 items on medication necessity and 5 items on medication concern. A 5-point Likert scale was used for each item, ranging from 1 (strongly disagree) to 5 (strongly agree). The overall score ranged from 5 to 25, with higher values indicating stronger beliefs about necessity and concerns. Additionally, a necessity-concerns differential (NCD) was derived by subtracting the concerns score from the necessity score, producing a range of −20 to 20. This metric served as an indicator of the balance between perceived benefits and concerns, representing an individualized cost-benefit assessment of medication use [26]. The simplified Chinese version demonstrated good reliability, with Cronbach α values of 0.826 (necessity) and 0.820 (concern) and intraclass correlation coefficients of 0.784 and 0.732, respectively [23].

### mVTEA-Facilitated Patient-Centered VTE Management

When designing the mVTEA, we took into account the fact that the long-term management of VTE is multifaceted, requiring individualized patient assessment and a nuanced approach to therapy. Therefore, we proposed the ABCDEF pathway for integrated management of VTE to complement mVTEA-assisted patient-centered management, thus minimizing the risk of VTE and its complications and ensuring patient safety and quality of life: (A) appropriate antithrombotic management; (B) bleeding risk management; (C) complication monitoring; (D) digital health management; (E) exercise and rehabilitation; and (F) facilitate the management of risk factors and comorbidities [16].

**Figure 1.**
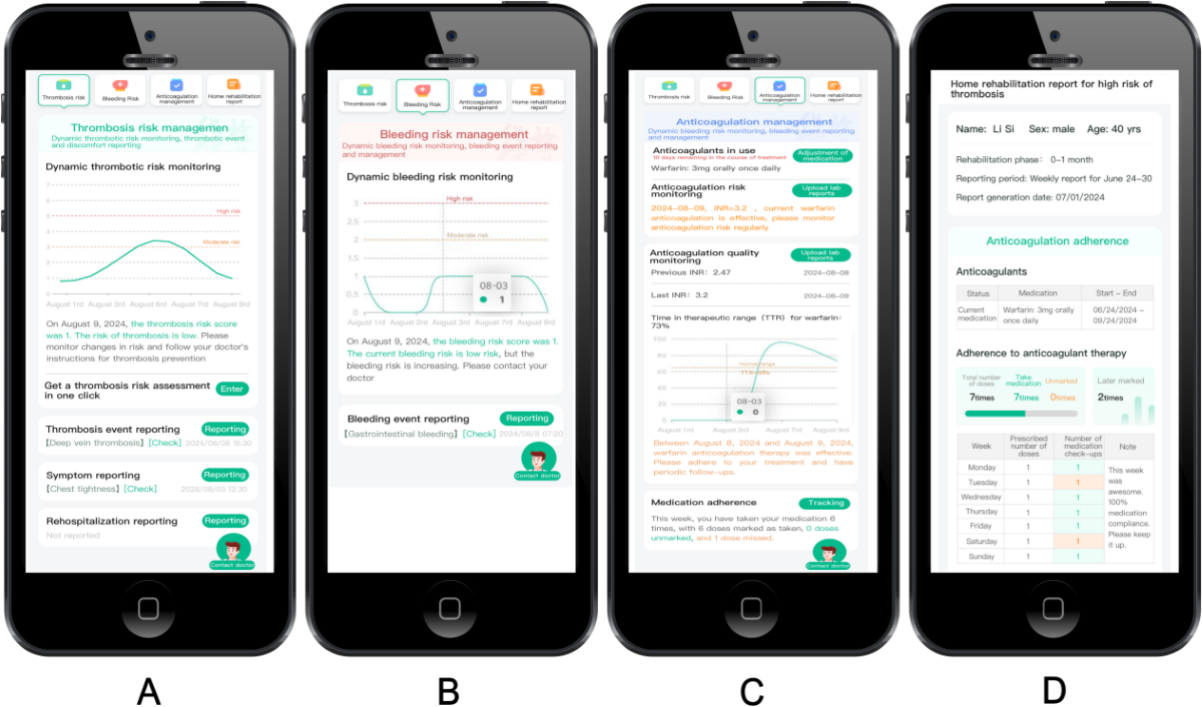
The core functional modules of the smart technology–assisted, patient-centered care mobile health app for venous thromboembolism management: (**A**) thrombosis risk assessment, (**B**) bleeding risk assessment, (**C**) anticoagulation management, and (**D**) home rehabilitation report.

The mVTEA was designed as a patient-side and physician-side interface. The core functions of the patient interface were four modules: thrombosis risk assessment, bleeding risk assessment, anticoagulation management, and home rehabilitation report (Figure 1).

The Thrombosis and Bleeding Risk module provides dynamic monitoring of a patient’s risk for thrombosis and bleeding. Automated risk assessments were initiated when patients reported symptoms or adverse events or uploaded relevant test results. Additionally, patients might complete a thrombosis risk self-assessment form. Based on the individualized risk profile, the mVTEA patient interface generates personalized recommendations for VTE prevention and treatment. These risk assessment outcomes were also transmitted to the mVTEA physician interface for further evaluation and feedback from thrombosis specialists.

The Anticoagulation Management module was structured into four key components: anticoagulants in use, anticoagulation risk monitoring, anticoagulation quality monitoring, and medication adherence. For anticoagulation risk and quality monitoring, patients could upload test results related to coagulation or liver or kidney function. These results were synchronized with the physician interface, where thrombosis specialists performed additional evaluations and interventions as needed. The medication adherence feature allowed patients to log anticoagulant administration through a digital “punch card” system, which tracked and displayed their adherence history.

The Home Rehabilitation Report module outlined the patient’s home-based rehabilitation plan, including the duration of anticoagulant therapy, historical anticoagulant usage, detailed adherence records, and VTE prevention and management recommendations. This report was automatically generated on a weekly and monthly basis for continuous monitoring and support.

Additionally, the mVTEA app offered daily medication reminders. At the end of each week, the app would provide feedback on the number of days the anticoagulant was taken as prescribed. The app also issued alerts when there were changes in thrombosis or bleeding risk assessments and monitored anticoagulation quality, such as the international normalized ratio for patients on warfarin. Patients could access their electronic health records at any time. When necessary, thrombosis specialists could deliver patient health education, including motivational interviewing, or conduct online consultations via the mVTEA doctor-patient communication module, which supported text, photo, and voice interactions. Outpatient appointments could also be arranged as needed through mVTEA.

To ensure the privacy and security of medical data during transfer from the hospital to the patient app, the mVTEA app used a novel QR code–based Security Transmission algorithm using Avro and Byte Pair Encoding (QRST-AB) [27]. The algorithm processes a deidentified dataset containing essential clinical information in a structured JSON format, including medical history, laboratory tests, and imaging results, while excluding all direct personal identifiers. This guarantees that the transmitted data alone cannot be linked to a specific individual. The QRST-AB framework integrates the ChaCha20 stream cipher for encryption and a BLAKE3 hash-based mechanism for authentication, which verifies the user’s identity against a digital fingerprint of the QR code content. Data are transmitted via offline QR codes, eliminating the risk of network exposure. The security of the system is further underpinned by proprietary Avro serialization schemas and a Byte Pair Encoding tokenizer. These core components are synchronized between the encoder and decoder through secure offline channels, rendering the data structure and content uninterpretable to third parties, thereby collectively ensuring comprehensive data protection.

### Outcome Measures

The primary end point was the proportion of patients with perfect adherence to the prescribed anticoagulant regimen at the 1-month follow-up. Perfect adherence was defined as 100% concordance between the patient’s self-reported medication use (verified against pharmacy refill records and remaining medication packaging) and the prescribed regimen in terms of medication type, dosage, and frequency. Patients were classified as having “good adherence” (fully adherent) if no deviations were identified and “poor adherence” if any deviation (eg, missed doses, incorrect dosage, or premature discontinuation) was confirmed.

The secondary end points included: (1) the interactive anticoagulant therapy reminder check-in rate via mVTEA, calculated as the ratio of completed check-ins for prescribed anticoagulant doses to the total number of prescribed doses; (2) clinical safety and efficacy outcomes, including VTE events, major bleeding events defined by the International Society on Thrombosis and Haemostasis criteria, VTE-related hospitalizations, VTE-related rehospitalizations, and all-cause deaths. Hospitalization due to the new-onset VTE would be documented as VTE-related hospitalizations, while rehospitalization due to VTE recurrence, progression, or complications arising from VTE treatment would be classified as VTE-related rehospitalizations. All-cause death referred to any death occurring during the study period, regardless of the cause.

### Data Collection

Patient demographic and clinical information would be extracted from the hospital information system. Baseline medication adherence and beliefs were assessed using the Chinese versions of MMAS-8 and BMQ-Specific prior to discharge. The primary measure of anticoagulation adherence at 1-month follow-up was determined through a comprehensive approach involving structured telephone follow-ups, outpatient clinic visits, and the physician-patient communication module within the mVTEA app. During these interactions, researchers recorded the current medication regimen, verified discrepancies against discharge prescriptions, documented missed doses, and confirmed therapy continuation. Whenever possible, adherence was objectively verified against pharmacy refill records or remaining medication packaging. The secondary end points were also collected during these follow-up assessments. Clinical safety events were documented based on patient reports and verified against medical records. Additionally, the interactive check-in rate via the mVTEA was automatically logged to assess patient engagement with the app.

### Ethical Considerations

The study protocol was approved by the Independent Ethics Committee of the Sixth Medical Center of the Chinese People’s Liberation Army General Hospital (approval HZKY-PJ-2022‐21). All procedures adhered to the principles of the Declaration of Helsinki. All participants provided written informed consent for study participation and use of the mVTEA. They were explicitly informed of their right to withdraw from the study at any time without penalty. No financial compensation, material incentives, or other forms of remuneration were offered to participants for enrollment or study participation. Participation in the study was entirely voluntary, and refusal or withdrawal did not influence access to standard clinical care or follow-up management. To ensure data privacy and security, all patient data were deidentified and assigned a unique study code during data processing. The collected data were securely stored and managed by designated personnel, accessible only to authorized researchers for the sole purpose of this study. No data were disclosed to third parties.

### Statistical Analysis

This study was a pilot study to evaluate the feasibility and preliminary efficacy of the mVTEA in improving short-term anticoagulation adherence. The calculation of sample size for this pilot study was determined using the method proposed by Viechtbauer et al [28]. Assuming the minimum detectable change in medication adherence is 0.1, under the requirement of achieving a 95% confidence interval, the minimum sample size for this study was calculated to be 29 people.

All statistical analyses were performed using SPSS (version 20.0; IBM Corp), with continuous variables presented as mean (SD) or median (IQR) and categorical variables as counts and percentages. Baseline medication adherence and beliefs were assessed using the Chinese versions of the MMAS-8 and BMQ-Specific. Based on baseline MMAS-8 scores, participants were categorized into good, moderate, and poor adherence groups, and the Kruskal-Wallis *H* test was used to analyze differences in MMAS-8 item scores among these groups. If a significant overall effect was observed (*P*<.05), post hoc pairwise comparisons were conducted using the Dunn test with Bonferroni correction to determine specific differences between groups. Based on the NCD calculated from BMQ-Specific scores, participants were divided into High-BMQ (NCD>0) and Low-BMQ (NCD ≤0) groups, and the Mann-Whitney *U* test was used to compare BMQ-Specific item scores between them. Additionally, the *χ*^2^ test (or Fisher exact test) was used to compare the differences in mVTEA medication check-in rates across the three MMAS-8 adherence groups and the two NCD groups. Statistical significance was set at *P*<.05.

## Results

### Participant Characteristics

**Figure 2.**
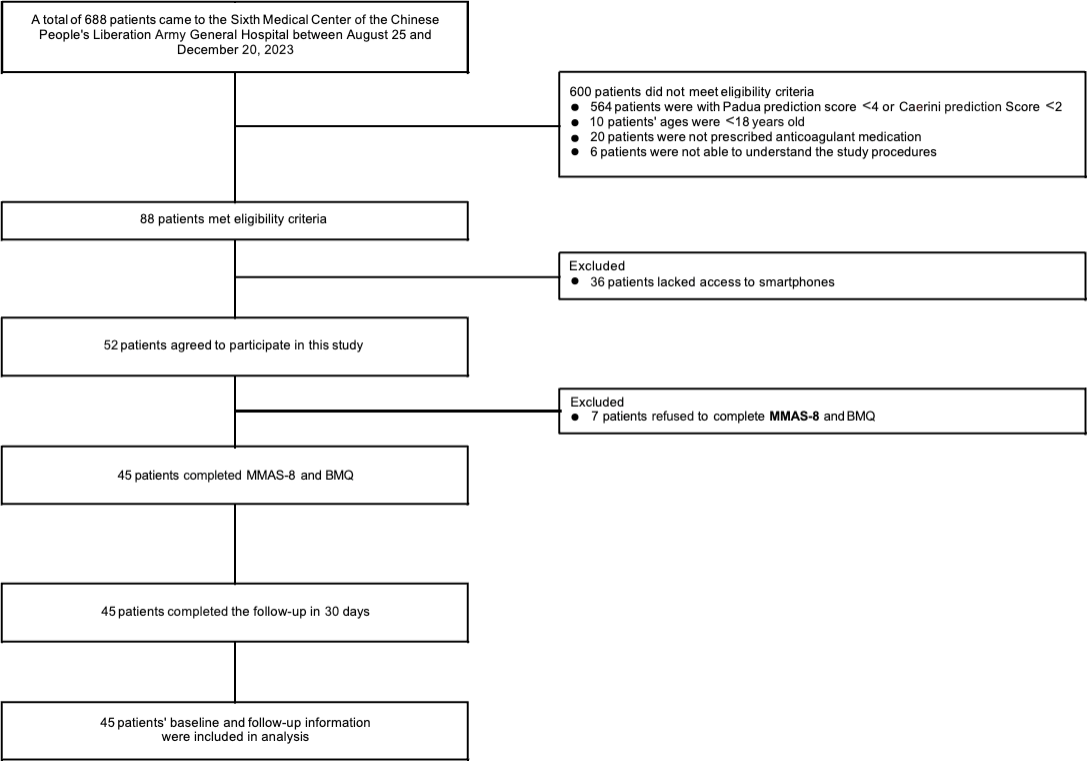
Study flowchart. BMQ: Beliefs about Medicines Questionnaire; MMAS-8: 8-item Morisky Medication Adherence Scale.

From August 25 to December 20, 2023, a total of 45 patients were enrolled, among which 87% (39/45) were diagnosed with VTE. The flowchart is shown in Figure 2. The mean patient age was 60.80 (SD 15.20) years and 36% (16/45) of the patients were females. The detailed patient characteristics are illustrated in Table 1.

**Table 1.** Baseline demographic and clinical characteristics of the study population.

Item	Patients (N=45）
Age (y), mean (SD)	60.80 (15.20)
Female, n (%)	16 (36)
Risk of VTE^a^, n (%)	
Medium	2 (4)
High	4 (9)
VTE patients, n (%)	
DVT^b^	18 (40)
PE^c^	11 (24)
DVT and PE	10 (22)
Comorbidities, n (%)	
Hypertension	19 (42)
History of cancer	2 (4)
Diabetes	6 (13)
Previous stroke	2 (4)
Atrial fibrillation	3 (7)
Valvular heart disease	1 (2)
Others	12 (27)

a VTE: venous thrombosis.

b DVT: deep venous thrombosis.

c PE: pulmonary embolism.

### Medication Adherence at Baseline

**Figure 3.**
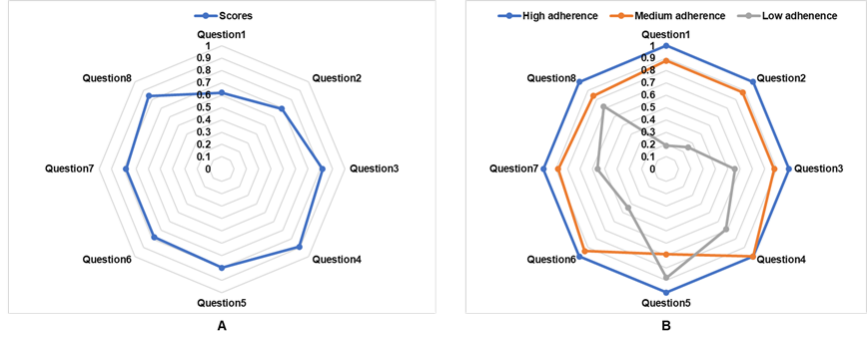
Distribution of baseline 8-item Morisky Medication Adherence Scale scores among the study population: (A) overall distribution and (B) stratified by adherence level.

Tables2  3 summarize the baseline MMAS-8 scores, revealing suboptimal medication adherence among participants. Specifically, only 29% (13/45) of patients exhibited good adherence, while the majority (71%, 32/45) demonstrated medium or poor adherence levels. The predominant factors contributing to this low adherence were identified as “Sometimes forget to take their medication” (Question 1) and “People sometimes forget to take their medications for reasons other than forgetting” (Question 2). Notably, these same factors were consistently reported among poorly adherent patients. Figure 3 illustrates the baseline distribution of MMAS-8 scores across the study cohort, along with the score distributions stratified by different adherence categories.

**Table 2.** Baseline medication adherence scores measured by the 8-item Morisky Medication Adherence Scale (MMAS-8) for the study population.

MMAS-8^a^ item	Scores, mean (SD)
Q1. Do you sometimes forget to take your medication?	0.62 (0.49)
Q2. People sometimes forget to take their medications for reasons other than forgetting. Thinking over the past two weeks, were there any days when you did not take your medication?	0.69 (0.47)
Q3. Have you ever cut back or stopped taking your medication without telling your doctor, because you felt worse when you took it?	0.82 (0.39)
Q4. When you travel or leave home, do you sometimes forget to bring your medication?	0.89 (0.32)
Q5. Did you take your medication the last time you were supposed to take it?	0.80 (0.41)
Q6. When you feel like your symptoms are under control, do you sometimes stop taking your medication?	0.78 (0.42)
Q7. Taking medication every day is a real inconvenience for some people. Do you ever feel hassled about sticking to your treatment plan?	0.78 (0.42)
Q8. How often do you have difficulty remembering to take all your medications?	0.84 (0.22)
Total scores	6.24 (1.80)

a MMAS-8: 8-item Morisky Medication Adherence Scale.

**Table 3.** Baseline medication adherence scores measured by the 8-item Morisky Medication Adherence Scale (MMAS-8) stratified by adherence levels within the study population.

Item of MMAS-8^a^	High adherence (n=13), mean (SD)	Medium adherence (n=16), mean (SD)	Low adherence (n=16), mean (SD)	*P* value
Question 1	1.00 (0.00)	0.88 (0.34)	0.19 (0.40)	<.001
Question 2	1.00 (0.00)	0.88 (0.34)	0.25 (0.45)	<.001
Question 3	1.00 (0.00)	0.94 (0.25)	0.56 (0.51)	.003
Question 4	1.00 (0.00)	1.00 (0.00)	0.69 (0.48)	.007
Question 5	1.00 (0.00)	0.63 (0.50)	0.81 (0.40)	.045
Question 6	1.00 (0.00)	0.94 (0.25)	0.44 (0.51)	<.001
Question 7	1.00 (0.00)	0.81 (0.40)	0.56 (0.51)	.02
Question 8	1.00 (0.00)	0.84 (0.27)	0.69 (0.25)	<.001

a MMAS-8: 8-item Morisky Medication Adherence Scale.

### Belief About Taking Anticoagulants at Baseline

**Figure 4.**
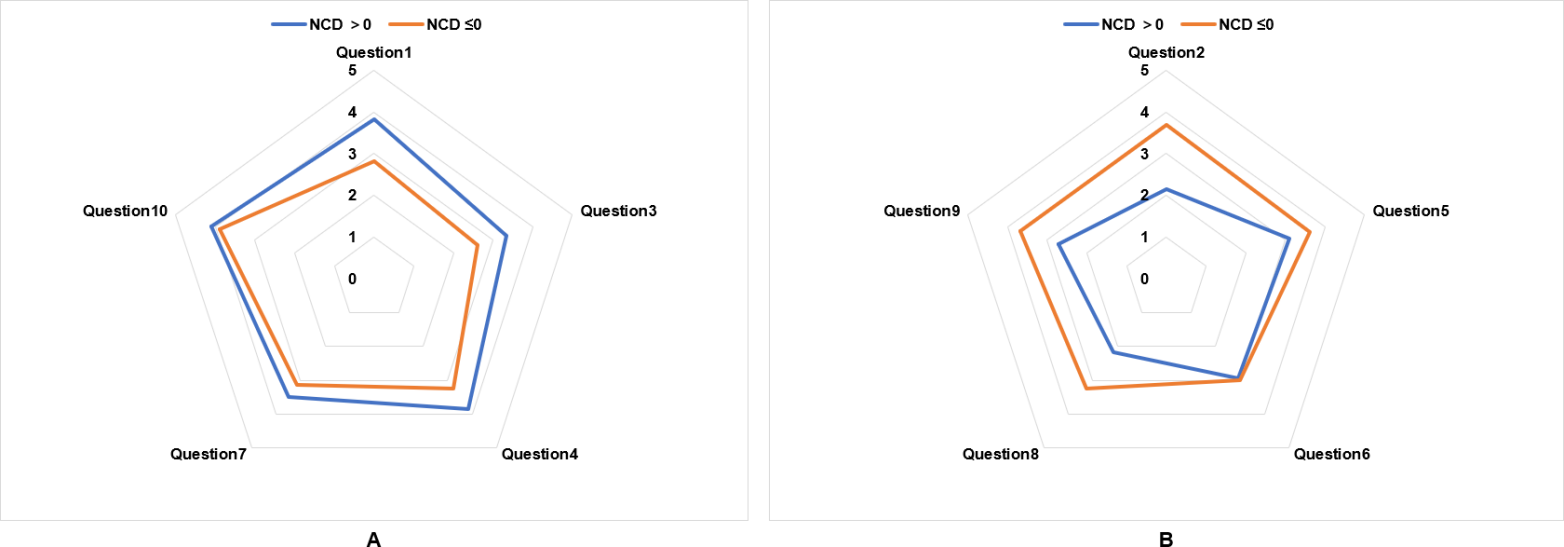
Differences in baseline Beliefs about Medicines Questionnaire–Specific (A) necessity and (B) concern item scores between patients with positive (NCD >0) and nonpositive (NCD ≤0) NCDs among the study population. NCD: necessity-concerns differential.

Tables4  5 present the BMQ-Specific scores for individual questionnaire items. Among the 45 participants who completed the baseline BMQ-Specific assessment, the necessity score for anticoagulant therapy significantly exceeded the concern score (17.58, SD 2.52 vs 14.56, SD 3.34; *P*<.001). The primary factor contributing to insufficient perceived necessity was the statement “Without my anticoagulants, I would be very ill” (Question 3), while the predominant concern was reflected in the response to “I sometimes worry about the long-term effects of my anticoagulants” (Question 5). In the Low-BMQ group (NCD ≤0), inadequate belief in treatment necessity was primarily associated with responses to “Currently, my health depends on anticoagulants” (Question 1) and Question 3, whereas most of the concern-related items demonstrated elevated scores (Figure 4).

**Table 4.** Baseline anticoagulant beliefs measured by the Beliefs about Medicines Questionnaire–Specific for the study population.

Characteristics classification	Scores, mean (SD)
BMQ^a^-Specific necessity	17.58 (2.52)
Q1. Currently, my health depends on anticoagulants.	3.47 (0.70)
Q3. Without my anticoagulants, I would be very ill.	3.09 (0.73)
Q4. My life would be impossible without my anticoagulants.	3.64 (0.74)
Q7. My health in the future will depend on my anticoagulants.	3.36 (0.74)
Q10. My anticoagulants prevent my condition from worsening.	4.02 (0.50)
BMQ-Specific concern	14.56 (3.34)
Q2. Having to take my anticoagulants worries me.	2.69 (1.26)
Q5. I sometimes worry about the long-term effects of my anticoagulants.	3.29 (0.84)
Q6. My anticoagulants are a mystery to me.	2.96 (0.98)
Q8. My anticoagulants disrupt my life.	2.56 (0.89)
Q9. I sometimes worry about being too dependent on my anticoagulants.	3.07 (1.01)
BMQ-Specific NCD^b^	3.02 (4.60)

a BMQ: Beliefs about Medicines Questionnaire.

b NCD: necessity-concerns differential.

**Table 5.** Differences in baseline Beliefs about Medicines Questionnaire (BMQ)–Specific necessity and concern item scores between the High-BMQ group and the Low-BMQ group.

Item of BMQ-Specific, mean (SD)	High-BMQ^a^ group (NCD >0)^b^, n=29	Low-BMQ group (NCD ≤0), n=16	*P* value
BMQ-Specific necessity			
Question 1	3.83 (0.54)	2.81 (0.98)	<.001
Question 3	3.34 (0.72)	2.63 (0.50)	.001
Question 4	3.86 (0.64)	3.25 (0.77)	.005
Question 7	3.48 (0.63)	3.13 (0.89)	.19
Question 10	4.10 (0.49)	3.88 (0.50)	.14
BMQ-Specific concern			
Question 2	2.14 (0.95)	3.68 (1.14)	<.001
Question 5	3.10 (0.82)	3.62 (0.81)	.10
Question 6	2.93 (1.10)	3.00 (0.73)	.92
Question 8	2.17 (0.66)	3.25 (0.86)	<.001
Question 9	2.72 (1.03)	3.69 (0.50)	.002

a BMQ: Beliefs about Medicines Questionnaire.

b NCD: necessity-concerns differential.

### The Impact of mVTEA on Anticoagulation Adherence and Adverse Events

The results showed 100% adherence to the prescribed anticoagulants for all patients at the 1-month follow-up. The check-in rate for interactive anticoagulant therapy reminders through mVTEA was 80% (36/45). Table 6 shows those participating in check-ins during the 1-month follow-up (n=36) had significantly better medication adherence and beliefs about taking anticoagulants. All patients in the no check-in group (n=9) had a negative NCD, compared to only 19% (7/36) of patients in the check-in group. No VTE events, major bleeding, or other adverse outcomes were reported at 1-month follow-up.

**Table 6.** Comparison of baseline medication adherence and anticoagulant beliefs between patients with and without engagement in the mobile health app for patient-centered venous thromboembolism management check-ins during the 1-month follow-up among the study population.

	Check-in (n=36)	No check-in (n=9)	*P* value
MMAS-8^a^, n (%)			
High	13 (36)	0 (0)	.09
Medium	11 (31)	5 (56)	
Low	12 (33)	4 (44)	
BMQ^b^-Specific, n (%)			<.001
NCD^c^ ≥0	29 (81)	0 (0)	
NCD <0	7 (19)	9 (100)	

a MMAS-8: 8-item Morisky Medication Adherence Scale.

b BMQ: Beliefs about Medicines Questionnaire.

c NCD: necessity-concerns differential.

## Discussion

### Principal Findings

This pilot study assessed the feasibility of implementing the mVTEA, a patient-centered mobile health intervention designed to support anticoagulation management among patients with VTE or at moderate-to-high risk of VTE. Rather than demonstrating clinical efficacy, the findings primarily indicate operational feasibility and provide early behavioral signals relevant to planning a definitive randomized trial. A 100% adherence rate was recorded at 1 month, accompanied by an 80% engagement rate with the app’s check-in function. These results should be interpreted as preliminary indicators of acceptability rather than evidence of intervention effectiveness.

Several factors likely contributed to the high adherence observed in this study. First, the baseline assessment revealed that our cohort possessed relatively strong medication beliefs, with a positive NCD indicating that patients perceived the benefits of anticoagulation as outweighing the risks. Second, the 1-month observation period captured the acute phase of treatment, a period typically characterized by high patient motivation. Third, the exclusion of patients unable to use smartphones ensured a cohort with high eHealth literacy, potentially creating a selection bias toward individuals more receptive to digital interventions.

Critically, the high adherence rate may also reflect the direct impact of the mVTEA intervention itself. Unlike passive reminders, the mVTEA uses a conversational interface and interactive modules to provide personalized feedback, actively engaging patients in self-management [16]. This real-time engagement likely reinforced disease awareness and heightened the perceived necessity of therapy, creating a positive feedback loop that encouraged strict adherence. Therefore, the 100% adherence may represent a synergistic effect of favorable baseline characteristics and the potent behavioral support provided by the technology.

The 80% check-in rate underscores the value of patient-centered design in overcoming common barriers such as forgetfulness. Moreover, the association between app engagement and favorable medication beliefs suggests that mVTEA functions as an adjunct to reinforce treatment necessity. While conducted within the Chinese health care system, these findings have broader relevance. The mVTEA model—integrating digital risk assessment with patient engagement—could be adapted for health care systems with limited outpatient resources by reducing the burden of routine follow-ups. The scalability of such tools suggests a viable pathway to support anticoagulation management in diverse settings, although the infrastructure required for digital access remains a prerequisite.

The present findings contribute to the growing evidence supporting mHealth interventions in cardiovascular disease management. Previous research has consistently demonstrated the effectiveness of smartphone apps in improving medication adherence. Specifically, Cao et al [29] and Jiang et al [30] reported that the Alfalfa app significantly improved anticoagulation control and reduced adverse events in warfarin therapy. These findings were corroborated by Talboom-Kamp et al [31], who established the safety and reliability of eHealth platforms for oral anticoagulation self-management. In contrast to prior studies, our investigation focuses on a more heterogeneous VTE population receiving various anticoagulant therapies, while incorporating a dedicated VTE protocol featuring personalized risk assessment and direct clinician-patient interaction. The mAFA trials [12,32] previously demonstrated the efficacy of an integrated mHealth approach incorporating the ABC pathway for atrial fibrillation, showing improved treatment adherence and clinical outcomes. Our study adapted this patient-centered, integrated model to the distinct clinical context of VTE management. Additionally, our results are consistent with existing literature documenting mHealth’s potential to enhance patient engagement and overcome health care access barriers, as demonstrated by high participation rates in mobile-based cardiac rehabilitation programs [33, 34]. These findings underscore the need for large-scale randomized controlled trials to evaluate whether the observed improvements in adherence translate into reduced VTE recurrence and bleeding complications—critical clinical outcomes that remain insufficiently explored in contemporary mHealth research.

This study has several limitations. First, the single-arm design and lack of a control group preclude causal inference regarding the effectiveness of the intervention. The sample size was calculated solely for feasibility, rendering the study underpowered to detect efficacy differences. Second, the 100% adherence rate, while encouraging, must be interpreted with caution. Adherence was primarily assessed via self-report and medication logs, which are susceptible to recall and social desirability biases, compounded by the potential Hawthorne effect. Future large-scale randomized controlled trials should incorporate objective measures (eg, pharmacy refill data) and longer follow-ups to assess sustainability. Third, while the Chinese versions of MMAS-8 and BMQ-Specific are widely used [20-25], their specific validation in VTE populations is limited, and the scales’ susceptibility to bias warrants caution when interpreting psychological determinants. Finally, the exclusion of patients without smartphones or with low digital literacy introduces selection bias and likely inflated adherence estimates. The excluded population is often older, has multiple comorbidities, and is precisely the group that may benefit most from adherence support. Consequently, the generalizability of these findings to the broader VTE population is limited. Future iterations of the mVTEA must address this gap, perhaps through simplified interfaces, family-assisted modules, or hybrid digital-analog models to ensure equitable access.

### Conclusion

This pilot study demonstrates the feasibility and acceptability of the mVTEA to support anticoagulation management among patients with VTE or at risk of VTE and offers preliminary behavioral insights to inform the design of future trials. Larger, randomized studies with extended follow-up and objective adherence measures are required to evaluate long-term effectiveness. Addressing digital inequities will be essential to ensure that mHealth-supported anticoagulation management is accessible and beneficial to all patient groups.

## Supplementary material

10.2196/75508 Multimedia Appendix 1 The Chinese version of the 8-item Morisky Medication Adherence Scale (MMAS-8).

10.2196/75508 Multimedia Appendix 2 The simplified Chinese version of the Beliefs about Medicines Questionnaire–Specific (BMQ-Specific).

10.2196/75508 Checklist 1 CONSORT checklist.

## Abbreviations

BMQ-Specific: Beliefs about Medication Questionnaire–Specific
CONSORT: Consolidated Standards of Reporting Trials
mHealth: mobile health
MMAS-8: 8-item Morisky Medication Adherence Scale
mVTEA: mHealth app for managing VTE
NCD: necessity-concerns differential
PE: pulmonary embolism
QRST-AB: QR code–based Security Transmission algorithm using Avro and Byte Pair Encoding
VTE: venous thromboembolism
