# Supplementary material for: Smart Technology–Assisted Patient-Centered Management in Venous Thromboembolism: Pilot Study on Anticoagulation Adherence
Source: JMIR Form Res. 2026 Apr 2;10:e75508. doi: 10.2196/75508 (PMC13045778; doi:10.2196/75508)
Supplement: Multimedia Appendix 1 [file formative-v10-e75508-s001.pdf]

adherence.

您提到因为健康原因正在服用一些药物。这里有一些与服药行为相关的问题，而我们对您的个人经历很感兴趣。所以请您根据自己的服药经历对以下每一个问题做出回答，没有正确或错误的答案，如实回答即可。。
（请在下面回答）

MMAS-8 (Simplified Chinese) 服药依从性问卷

	Yes 是	No 否
1. Do you sometimes forget to take your medication(s)?<br>您是否有时会忘记服药？		
2 .Over the past two weeks, were there any days when you did not take your medicine(s)?<br>在过去的两个星期内，您忘记过服药吗？		
3. Have you ever cut back or stopped taking your medication(s) without telling your doctor because you felt worse when you took it?<br>当服药后感觉身体更糟时，您是否曾在没有告诉医生的情况下就自行减少药量或停药？		
4. When you travel or leave home, do you sometimes forget to bring along your medication(s)?<br>当你因旅行或其他原因离家外出时，您是否有时会忘记随身携带药物？		
5. Did you take your medicine(s) yesterday?<br>您昨天服药了吗？		

6. When you feel like your disease is under control, do you sometimes stop taking your medicine(s)?<br>当您觉得疾病得到控制时，您是否曾停药？					
7. Taking medication(s) everyday is a real inconvenience for some people. Do you ever feel hassled about sticking to your illness treatment plan?<br>对一些人来说，每天服药很不方便，您是否曾觉得坚持治疗方案（遵医嘱按时服药）很麻烦？					
	a	b	c	d	e
8. How often do you have difficulty remembering to take all your medication(s)?<br>您忘记服药的频率是？					

从不/很少……………..….a
偶尔………………………b
有时………………………c
经常………………………d
每次………………………e

Never/Rarely……………………………………....a
Once in a while…………………………………b
Sometimes……………………………………....c
Usually…………………………………………….d
All the time……………………………………...e

MMAS© www.adherence.cc
