# Supplementary material for: Smart Technology–Assisted Patient-Centered Management in Venous Thromboembolism: Pilot Study on Anticoagulation Adherence
Source: JMIR Form Res. 2026 Apr 2;10:e75508. doi: 10.2196/75508 (PMC13045778; doi:10.2196/75508)
Supplement: Multimedia Appendix 2 [file formative-v10-e75508-s002.pdf]

- 服药信念（中文版BMQ-Specific问卷）
    - 维度一：必要性信念（5个条目）
    - 维度二：顾虑信念（5个条目）
    - 分为必要性信念（包括条目１、３、４、７、１０）和顾虑信念（包括条目２、５、６、８、９），分别用于评估患者服用抗凝药物的积极态度和消极态度。
    - 采用Likert 5 等级评分法，１～５分分别表示非常不同意、不同意、不确定、同意和非常同意。
    - 必要性信念与顾虑信念得分之差的范围是－２０～＋２０分，该得分反映了患者对服用抗凝药物治疗的成本效益分析，若得分差值为负值，表示患者对药物的担心顾虑超过了对服药必要性的信念，反之亦然。
    -

| 服药信念量表—抗凝药<br>下表是用来评估您的用药观点及态度的情况，请根据您的真实情况在相应选项前进行打“√” | | | | |
|---|---|---|---|---|
| **1.我当前的健康状况依赖于服用抗凝药物** | | | | |
| □非常不同意 | □不同意 | □不确定 | □同意 | □非常同意 |
| **2.得病后必须坚持服用抗凝药物让我感到很烦恼** | | | | |
| □非常不同意 | □不同意 | □不确定 | □同意 | □非常同意 |
| **3.如果不服用抗凝药物，我将无法生存** | | | | |
| □非常不同意 | □不同意 | □不确定 | □同意 | □非常同意 |
| **4.如果不服用抗凝药物，我的病情会很重** | | | | |
| □非常不同意 | □不同意 | □不确定 | □同意 | □非常同意 |
| **5.我有时会担心抗凝药物的长期效果** | | | | |
| □非常不同意 | □不同意 | □不确定 | □同意 | □非常同意 |
| **6.抗凝药物对我来说很神秘** | | | | |
| □非常不同意 | □不同意 | □不确定 | □同意 | □非常同意 |
| **7.我未来的健康状况取决于抗凝药物的服用** | | | | |
| □非常不同意 | □不同意 | □不确定 | □同意 | □非常同意 |
| **8.抗凝药物扰乱了我的生活** | | | | |
| □非常不同意 | □不同意 | □不确定 | □同意 | □非常同意 |
| **9.我有时担心自己过于依赖抗凝药物** | | | | |
| □非常不同意 | □不同意 | □不确定 | □同意 | □非常同意 |
| **10.抗凝药物可以帮助我防止疾病恶化** | | | | |
| □非常不同意 | □不同意 | □不确定 | □同意 | □非常同意 |

- 生活质量
    - VTE风险人群（EQ-5D-5L欧洲五维健康量表中文版）
        - 5个维度，5个问题/条目
    - DVT人群（VEINES-QOL/Sym静脉功能不全生活质量/症状量表中文版）
        - 4个维度，7个问题，25个条目
    - PE人群（PEmb-QoL肺栓塞生活质量量表中文版）
        - 6个维度，9个问题，40个条目
    - CTEPH人群（SF-36 v2简明生活质量量表第二版）
        - 8个维度，36个问题/条目
